# Hepatitis E Virus (HEV) Infection among Hemodialysis Patients from Southern Bulgaria

**DOI:** 10.3390/pathogens12101208

**Published:** 2023-09-30

**Authors:** Ani Kevorkyan, Elitsa Golkocheva-Markova, Ralitsa Raycheva, Vanya Rangelova, Radka Komitova, Mariya Atanasova, Valeri Tzekov, Tanya Kostadinova, Tsvetelina Chardakova

**Affiliations:** 1 Department of Epidemiology and Disaster Medicine, Faculty of Public Health, Medical University of Plovdiv, 4002 Plovdiv, Bulgaria; ani.kevorkyan@mu-plovdiv.bg; 2 National Reference Laboratory “Hepatitis Viruses”, National Center of Infectious and Parasitic Diseases, 1233 Sofia, Bulgaria; elmarkova2007@gmail.com; 3 Department of Social Medical and Public Health, Faculty of Public Health, Medical University of Plovdiv, 4002 Plovdiv, Bulgaria; r.raycheva@mu-plovdiv.bg; 4 Department of Infectious Diseases, Parasitology and Tropical Medicine, Faculty of Medicine, Medical University of Plovdiv, 4002 Plovdiv, Bulgaria; radka.komitova@mu-plovdiv.bg; 5 Department of Microbiology and Immunology, Faculty of Pharmacy, Medical University of Plovdiv, 4002 Plovdiv, Bulgaria; mariya.atanasova@mu-plovdiv.bg; 6 Laboratory of Virology, University Multi-Profile Hospital for Active Treatment “St. George”, 4002 Plovdiv, Bulgaria; 7 Section of Nephrology, Second Department of Internal Medicine, Faculty of Medicine, Medical University of Plovdiv, 4002 Plovdiv, Bulgaria; valeri.tsekov@mu-plovdiv.bg; 8 First Dialysis Services Bulgaria, 4000 Plovdiv, Bulgaria; tania_kost@abv.bg; 9 First Dialysis Services Bulgaria, 4400 Pazardzhik, Bulgaria; cveta4ar@abv.bg

**Keywords:** HEV prevalence, hemodialysis patients, predictors for seropositivity

## Abstract

Viral hepatitis B and C are widely recognized problems in hemodialysis (HD) patients. There have been increasing reports of the importance of the hepatitis E virus (HEV) in recent years, but the worldwide data on the seroprevalence of HEV among them are conflicting. The aim of the present study was to assess the seroprevalence of HEV in HD patients and to analyze the predictors of seropositivity. This study was conducted in 2020 in the central part of southern Bulgaria. A total of 225 patients were enrolled. An enzyme-linked immunosorbent assay for the determination of anti-HEV IgM/IgG was used. All patients were tested for the presence of HEV RNA. Anti-HEV IgM alone and anti-HEV IgG alone were found in 6 (2.7%) and 14 (6.2%) patients, respectively, and in 4 (1.8%) patients, they were found simultaneously. All patients were HEV RNA-negative. The overall HEV seroprevalence was 10.7% (24/225). The binominal logistic regression analysis of available predictors confirmed the role of vascular access and a duration of dialysis treatment over 5 years as predictors significantly associated with increased risk for HEV, and the consumption of bottled water with lower levels of HEV IgG seroprevalence among hemodialysis patients. The accumulated data are the basis for comparative analysis in subsequent trials in the same dialysis centers and for enhancing the range of screening markers used in this particular patient group.

## 1. Introduction

Prior to the identification of the hepatitis E virus (HEV), instances of extensive jaundice outbreaks transmitted by the fecal–oral route were exclusively linked to the hepatitis A virus (HAV). The actual incidence of hepatitis E has been obscured by this phenomenon. Currently, it has been observed that HEV is responsible for the predominant occurrence of acute hepatitis worldwide. Annually, approximately 20 million individuals are infected with this virus, but only 3 million cases exhibit clinical manifestations. Furthermore, the mortality rate associated with HEV infection is between 50,000 and 70,000 deaths [1]. The true prevalence of infected persons is likely underestimated due to the large frequency of asymptomatic cases and the variability in the sensitivity and specificity of diagnostic tests employed. Four decades since the year 1983, during which M. Balayan successfully isolated HEV [2], there remain unresolved inquiries within our understanding of this illness that necessitate additional study. The comprehensive set of animal reservoirs of infection in non-endemic regions, the efficacy of person-to-person transmission of the virus, the potential risk of infection, and the varying levels of chronification across several groups of immunosuppressed individuals are among the key factors under consideration.

One particular group that is of interest in the context of HEV is the population of patients who are undergoing maintenance hemodialysis (HD). In the English-language literature, two extensive review articles [3,4] have been published within the last decade. These studies provide significant insights into several facets of the subject matter, including the seroprevalence of HEV, probable modes of transmission, and factors that contribute to the risk of infection. The prevalence of HEV antibodies among HD patients exhibited an upward trend, rising from 6.6% during the period of 1994 to 2000 to 11.1% during the period of 2016 to 2020 [4]. The comprehensive understanding of risk variables associated with HEV infection among HD recipients remains incomplete. The topic of blood transfusion and hemodialysis in relation to HEV infection was a matter of dispute from January 1980 until September 2009 [3]. Nevertheless, current data extending to January 2020 have presented compelling evidence indicating that blood transfusion is linked to a roughly two-fold increase in the prevalence of HEV seropositivity among HD patients [4]. Hepatitis E in hemodialysis patients originating from Eastern European nations, particularly in the Balkans, has received limited attention in academic research [5,6,7,8]. Insufficient data referring specifically to Bulgaria are evident [9]. Simultaneously, there has been a consistent rise in the population of patients afflicted with end-stage renal disease (ESRD) undergoing HD therapy in Bulgaria, starting from the inception of the first HD facility in 1958 and persisting to the present day. By the conclusion of the year 2018, the total count of patients undergoing HD amounted to 7793 individuals [10]. These patients were distributed among 80 dialysis centers, with 60 of them being situated within state-/municipal-owned or multi-profile hospitals, and the remaining 20 being located in private multi-profile hospitals or free-standing dialysis centers [11].

The aim of the current study was to evaluate the seroprevalence of hepatitis E virus antibodies in HD patients residing in the southern region of Bulgaria, as well as to examine the predictors that may contribute to seropositivity.

## 2. Materials and Methods

### 2.1. Study Design and Population

**Figure 1:**
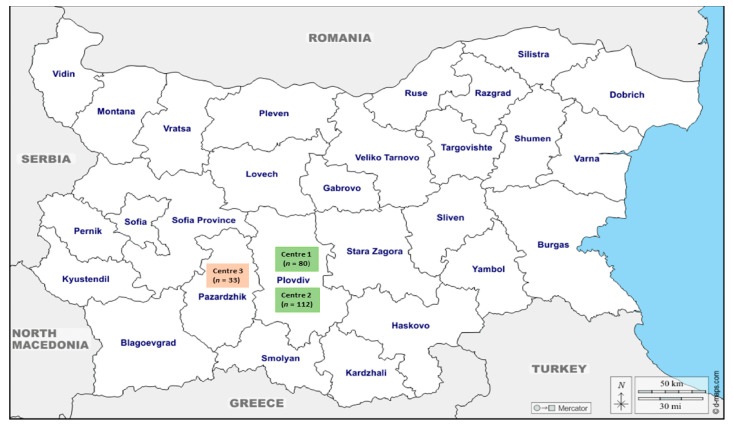
Distribution of the evaluated hemodialysis patients according to the administrative regions and centers.

The present prospective seroepidemiological study was conducted between January and August 2020 in three HD centers located in two neighboring administrative regions in the central part of southern Bulgaria (Plovdiv region and Pazardzhik region) (Figure 1). According to statistics gathered from reports published on the website of the National Health Insurance Fund, as of June 2023, there were a total of 578 patients receiving chronic HD treatment across both areas. These data encompass 12 dialysis centers.

This study utilized the convenience sampling technique to choose a sample of 225 patients who were undergoing 4 h dialysis treatments for chronic renal failure 3 times a week. The sample was divided among three centers: Center 1 (*n* = 80 patients), Center 2 (*n* = 112 patients), and Center 3 (*n* = 33 patients). Center 1, located in the Hemodialysis Department of the “St. George” University Hospital in Plovdiv, holds the distinction of being the oldest dialysis department in the city. Established in 1976, this center provides a total of 22 dialysis stations, spread across 3 halls. Center 2, known as “First Dialysis Services Bulgaria,” was established in the city of Plovdiv in 2013. It comprises 5 halls, housing a total of 30 dialysis stations. On the other hand, Center 3, also named “First Dialysis Services Bulgaria,” commenced operations in the city of Pazardzhik in 2019. It encompasses 3 halls, accommodating a total of 25 dialysis stations. Center 2 and Center 3 are privately owned dialysis facilities that operate independently. Each of the three centers has been specifically developed to provide treatment for those who are part of the adult population, defined as those who are 18 years of age or older.

Epi Info TM version 7.2.5 (CDC, Atlanta, GA, USA, 2020) was used for the calculation of the sample size. The population survey study formula was applied to achieve a 5% error margin and a 95% confidence level. The expected frequency was set to be 14.7%, the same as the prevalence of HEV among HD patients reported in a previous study conducted in Pazardzhik region [9]. The minimum number of participants was calculated to be 188, based on the 7793 overall population of hemodialysis patients, reported for Bulgaria in 2018 [10].

To facilitate the research objectives, a questionnaire (referred to as a questionnaire card) was designed. The survey comprised a total of 29 questions, which were categorized into 5 primary groups. The first group focused on demographic information such as gender, age, and place of residence. The second group gathered anamnestic data pertaining to the underlying kidney disease. The third group explored the medical history of the participants, including any instances of symptomatic hepatitis infection within their family. The fourth group examined intra-dialysis factors, encompassing the duration of dialysis treatment, vascular access, blood transfusion, and kidney transplantation. Lastly, the fifth group addressed extra-dialysis factors specific to HEV, including food and water intake, contact with animals, and any recent travel abroad within the past three months.

Inclusion criteria: all patients with ESRD on chronic HD treatment present at the time of the study in the respective dialysis centers.

Exclusion criteria:

- Patients transferred to another dialysis center during the study period;
- Transient dialysis patients (temporarily residing in the district due to travel, family visits, etc.);
- Patients with an acute medical indication requiring hemodialysis treatment;
- Patients undergoing peritoneal dialysis at home (they are not exposed to the same procedures and conditions as in the hemodialysis center).

### 2.2. Algorithm for Examining Patients

1. Familiarizing the patients with the purpose of the study and filling out an informed consent form. The collection of blood samples (5 mL of venous blood each) was conducted after the submission of informed consent.
2. Blood samples were immediately centrifuged (at 2000 rpm), and the serum was separated into two aliquots (one for anti-HEV antibodies detection and the second for HEV RNA detection) and stored at −80 °C until use.
3. Initially, all serum samples were tested for the presence of specific HEV antibodies (anti-HEV IgM and anti-HEV IgG).
4. Secondly, regardless of antibody results, all patients were tested for the presence of HEV RNA. According to the recommendations of the EASL (European Association for the Study of the Liver) [12], for the diagnosis of HEV infection (acute and chronic) in immunocompromised persons, it is recommended that serology and Nucleic Acid Amplification Testing (NAT) be used in combination, as a negative NAT does not exclude acute infection and serology is sometimes negative in immunosuppressed patients with chronic infection.
5. All specimens were negative for HEV RNA. Therefore, for the purpose of analysis, the samples were divided into two groups based on the presence of HEV antibodies: (1) HEV IgG seroprevalence (anti-HEV IgG alone); (2) and overall HEV seroprevalence, based on all positive samples regardless of the type of HEV antibody marker (anti-HEV IgM and/or anti-HEV IgG).
6. An additional examination for the presence of HBV (HBsAg) and HCV (anti-HCV) coinfection was performed.

#### 2.2.1. Serological Testing

Antibodies to HEV (anti-HEV IgM and IgG) were detected by ELISA (Euroimmun, Lübeck, Germany) according to the manufacturer’s instructions. HEV genotype 1 and 3 recombinant antigens were used. For both anti-HEV IgM and IgG ELISAs, the manufacturer reported a diagnostic accuracy of 100% for specificity and 97.8% for sensitivity. Samples tested were considered reactive for anti-HEV IgM at a signal/cut-off ratio (S/CO) of 1.1 or greater. For anti-HEV IgG, results were interpreted quantitatively in [IU/mL] according to the manufacturer’s instructions, with results ≥ 1.1 IU/mL considered positive. The anti-HEV ELISA IgG has a linearity range of 1 IU/mL to 22 IU/mL with a detection limit of 0.1 IU/mL. Considering the variations in sensitivity among diverse commercial diagnostic kits for detecting HEV antibodies [13], the diagnostic assay was chosen based on specificity instead of sensitivity. The Euroimmun was selected based on its established high specificity of 100% for anti-HEV IgM and 99.3% for anti-HEV IgG in patients lacking markers of HEV infection, as observed in our study population [14]. Additionally, other authors have utilized Euroimmun to assess the prevalence of anti-HEV among different patient groups [9,15,16,17].

In addition, all samples were tested by ELISA for HBsAg (DiaPro, Milan, Italy, and Biokit, Barcelona, Spain) and anti-HCV (DiaPro, Milan, Italy, and Biokit, Barcelona, Spain). To discriminate false-positive results from resolved HCV infection (in the case of anti-HCV (+) and HCV RNA (−) samples), confirmation by Western blot assay (HCV Blot 3.0, MP Diagnostics, Eschwege, Germany) was performed according to the manufacturer’s instructions. Only samples with an established anti-HCV (+) and HCV Blot (+) profile were considered positive.

#### 2.2.2. Quantification of the HEV RNA

HEV RNA was detected and quantified using the RealStar HEV RT-PCR Kit 2.0 (Altona Diagnostics, Hamburg, Germany) according to the manufacturer’s instructions. The minimum linear limit of quantitation of the kit is 10 IU/μL. Runs were considered valid if all controls met the quality standards and R2 ≥ 0.98 for the standard curve according to the manufacturer’s instructions. The analytical performance evaluation of the RealStar^®^ HEV RT-PCR Kit 2.0 was conducted by the manufacturer using the “1st World Health Organization International Standard for Hepatitis”. The analytical sensitivity of 0.20 IU/µL (95% CI: 0.12–0.45 IU/µL) was reported. Analytical specificity of the utilized oligonucleotides is guaranteed by means of sequence comparison analysis against publicly accessible sequences, ensuring detection of all relevant HEV genotypes.

### 2.3. Statistical Analysis

Quantitative variables were presented by mean and standard deviation (mean ± SD) or median (25th percentile; 75th percentile), based on the sample distribution. Qualitative variables were presented as numbers absolute/relative frequencies totals and percentages (*n*, %). The Kolmogorov–Smirnov test was applied to inform about the distribution of the patients sampled. Testing for an association between two nominal variables was performed using the Chi-square test. Based on the variables introduced in the model independent variables, identified by univariate analysis, the binominal logistic regression was used to estimate the probability that an observation falls into one of two categories of a dichotomous dependent variable. Odds ratios (ORs) and 95% confidence intervals (95% CIs) for the fixed effects were calculated. The *p*-values < 0.05 were considered statistically significant for all statistical tests. The systematization, processing, and analysis of the data were performed using SPSS v.26 for Windows (IBM Corp. Released 2019. Armonk, NY, USA: IBM Corp).

### 2.4. Ethical Statement

The patients’ participation in the study was voluntary, anonymous, and free of charge. This study was approved by the University Ethical Committee of the Medical University of Plovdiv (Protocol No. 2/8–9 April 2020). All patients provided written informed consent.

## 3. Results

The present seroepidemiological study included a sample of 225 patients from 3 hemodialysis centers, which accounted for roughly 38.9% (225/578) of the total number of hemodialysis patients in both regions (Figure 1).

The median age of the HD patients was 64 years (IQR 52.5; 71.5), as the youngest and the oldest were 24 and 95 years old, respectively. Out of the total enrollment of 225 subjects, 126 individuals, accounting for 56% of the sample, identified as male. This indicated a male-to-female ratio of 1.3:1. The median duration of hemodialysis (in months) was 48 (IQR 24; 72). The majority of patients were aged ≥50 years (80.4%, *n* = 181) and lived in a regional or smaller town (68.0%, *n* = 153). The three main causes contributing to the need for dialysis treatment, which had the highest occurrence rates among individuals, were autosomal dominant polycystic kidney disease (15.2%), chronic tubulointerstitial nephritis (15.2%), and chronic glomerulonephritis (14.7%). Arteriovenous fistula (AV fistula) was the preferred vascular access in 121 (53.8%) of the HD patients. Detailed information on HD patients (demographics, presence of intra- and extra-dialysis risk factors) is presented in Table 1.

Anti-HEV IgM alone was found in 6 (2.7%) individuals, and HEV IgG alone was found in 14 (6.2%). Additionally, four individuals (1.8%) demonstrated the presence of both HEV antibody markers concurrently. All HD patients were HEV RNA-negative. The evaluated patients were not characterized by the presence of symptoms for hepatitis. As described above, HEV IgG seroprevalence was determined in 14 (6.2%) of the HD patients. There was no statistically significant variation seen between the different sites (Table 2). The study found an overall seroprevalence of 10.7% (24/225) for HEV, with varying rates of 8.8%, 9.1%, and 12.5% observed at different centers (*p* > 0.05) (Table 2).

Further testing for HBV and HCV revealed the presence of HBsAg in 5.8% (*n* = 13) and anti-HCV in 6.7% (*n* = 15), with no statistically significant differences observed among the three sites (Table 2).

**Figure 2:**
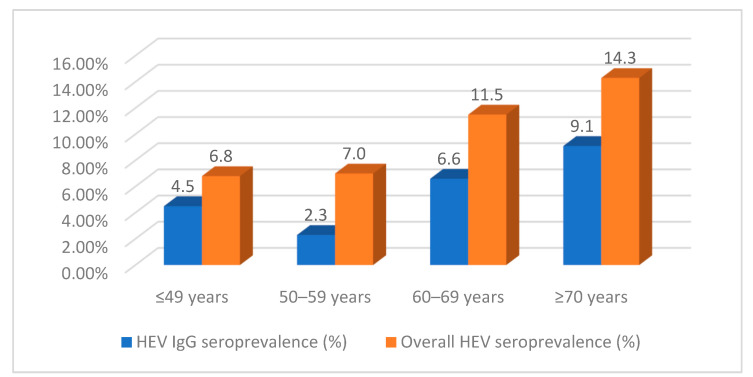
Age-specific tendency of HEV IgG seroprevalence group and overall HEV seroprevalence in HD patients.

In relation to the prevalence of HEV IgG and overall HEV, there was an observed increase in both seroprevalence rates, with age-specific prevalence showing an ascending trend. (Figure 2). Within the HEV IgG seroprevalence cohort, there was an observed increase in the prevalence rate from 4.5% among individuals aged 49 years or younger to 9.1% among those aged 70 years or over (z-test = 0.9, *p* = 0.353). Notably, a drop in prevalence was only observed within the age group of 50–59 years, with a rate of 2.3%. The decrease in the seroprevalence of HEV IgG in the age group of 50–59 years was not statistically significant when compared to the seroprevalence in the first age group of individuals aged ≤ 49 years (4.5%) (z-test = 0.6, *p* = 0.572). When examining the overall seroprevalence of HEV, it was seen that the positivity rates were relatively consistent at 6.8% and 7.0% in the first two age groups (≤49 years and 50–59 years), respectively. However, in those aged 70 years or above, the positivity rate increased to 14.3%. The observed rise in positive cases among different age groups did not reach statistical significance. Specifically, the percentage increase was 6.8% for individuals aged 49 years or under and 14.3% for those aged 70 years or over (z-test = 1.2, *p* = 0.215).

The findings of the univariate analysis are displayed in Table 3, which illustrates the distribution of patients based on their HEV profile, including HEV IgG seroprevalence versus HEV-negative, as well as overall HEV seroprevalence versus HEV-negative. Additionally, the table provides information on the patients’ primary demographic characteristics and risk factors.

A statistically significant difference was observed in the type of vascular access when comparing the HEV IgG seroprevalence group to the HEV-negative group, as determined by univariate analysis (Table 3).

A binomial logistic regression was applied to predict the probability that an observation falls into one of two categories of a dichotomous dependent variable—both for the overall HEV seroprevalence and the HEV IgG seroprevalence, respectively. No significant model for the overall HEV seroprevalence was established. The model fit for the positive (HEV IgG seroprevalence group) vs. negative result of HEV was statistically significant, Chi-square (14) = 23.776, *p* = 0.49, and explained 28.3% (Nagelkerke R2) of the variance in the HEV results and correctly classified 94.1% of cases (Table 4).

Overall, three significant variables were observed after the model fit. Hemodialysis duration over 5 years increased the likelihood of HEV infection versus less than a year of HD duration (OR = 6.67, 95% CI: 1.01–44.37). Patients with a permanent central venous catheter were more likely to be HEV-positive compared to patients with AV fistula (OR = 12.26, 95% CI: 2.19–68.73). This was the variable with the highest weight for the model (B = 2.54). Bottled water intake was associated with a lower likelihood of HEV IgG seroprevalence cases (OR = 0.20, 95% CI: 0.04–0.99).

## 4. Discussion

The present study focuses on a particular group, namely those diagnosed with end-stage renal disease who are undergoing regular hemodialysis treatment. Parenterally transmitted HBV and HCV are typically the primary areas of inquiry in these cases. Consequently, in accordance with the Bulgarian medical guidelines for hemodialysis treatment [18], it is imperative to conduct tests for HBsAg and anti-HCV as the main markers of HBV and HCV. Over the past few years, there has been a significant rise in the number of documented cases concerning the transmission of HEV in regions that were previously designated as non-endemic, with a particular focus on Europe [19,20,21,22,23,24,25]. The increase in cases, particularly among persons receiving hemodialysis treatment, has garnered the interest of experts, leading to additional research being conducted on this viral infection. The available data indicate a significant prevalence of locally acquired hepatitis E in Europe and other developed nations [20]. Furthermore, there is a possibility that in regions with low endemicity, hepatitis E may surpass hepatitis A and hepatitis B as the primary cause of hepatitis, owing to a decline in the incidence of the latter two [21]. In the year 2019, a formal registration process for the disease was launched in Bulgaria [26], marking 24 years since the initial confirmation of the first case of hepatitis E in the country by P. Teoharov in 1995 [27]. The existing data in Bulgaria cover multiple aspects of HEV infection, including examinations conducted among animal populations [28,29,30,31], a seroprevalence study conducted on the general population in a specific region of the country [32], analysis of the genotypic spectrum and phylogenetic relationships of isolates obtained from patients with acute hepatitis E [33,34], investigations on the presence of HEV in HIV-infected patients [35], and distinct regional studies focusing on the clinical manifestations of acute hepatitis E [36,37,38,39,40]. The aforementioned research predominantly depends on the identification of specific antibodies.

Our seroepidemiological study among HD patients is the first in the country to apply a combination of serological and molecular methods for HEV detection. The use of RT-PCR for HEV RNA detection allows for an accurate diagnosis and, when required, prompt monitoring and recommendation for therapeutic intervention.

The reported seroprevalence rates of HEV IgG (6.2%) and overall HEV (10.7%) are comparatively lower than the seroprevalence rate of 14.7% observed in another study conducted among hemodialysis patients in Pazardzhik, Bulgaria, in 2019 [9]. However, due to the utilization of three HEV markers (anti-HEV IgA, anti-HEV IgM, and anti-HEV IgG) to assess the seroprevalence level, a proper comparison of the data with our findings is not feasible. The available literature indicates that there is a significant variation in the seroprevalence of HEV IgG among individuals undergoing hemodialysis. In the context of Brazil, the lack of HEV IgG seroprevalence (0%) has been linked to the reduced immunological response observed in persons suffering from chronic renal failure [41]. On the other hand, it has been reported that there is a significantly high seroprevalence of 68.6% in southern Iran, suggesting the possible widespread presence of HEV among the general population [42]. This assertion is substantiated by the authors’ previous findings, which indicated a seroprevalence rate of 65.1% among individuals who were not undergoing hemodialysis. Prior studies undertaken in Greece at different time periods have shown significant discrepancies among dialysis centers across various locations of the nation [6,22]. Nevertheless, the findings of our study did not reveal any significant variations, as seen by the data presented in Table 2.

Without a doubt, the prevalence of HEV among HD patients is influenced by the local endemicity at the population level. This is because HD patients are exposed to similar environmental factors as the general population. The HEV IgG seroprevalence and the overall prevalence of HEV closely align with the prevalence rate of 9.04% observed in the general population of the Plovdiv region [32]. Previous research conducted in several countries has revealed a greater prevalence of infection among individuals undergoing HD compared to those who are considered healthy controls [23,43]. In Europe, Croatia has recorded one of the highest HEV rates at 27.9% among HD patients [5]. This rate is much higher compared to the lower prevalence of 7.1% among the general population in the country [16]. When assessing the extent of HEV seroprevalence, it is important to take into account many aspects that may have an impact, including the specific diagnostic tests employed [6,44], the duration of the study, the criteria for participant inclusion, and the dynamics of the antibodies themselves. In comparison to the persistent detection of anti-HAV IgG in the serum of individuals throughout their lives, the precise length of the immune response generated by anti-HEV IgG antibodies remains unknown [45]. Several researchers have recorded the persistent existence of anti-HEVIgG antibodies, with durations ranging from 6 months to 14 years [46]. All of the aforementioned factors have the ability to serve as predictors outside the confines of the dialysis unit, indicating that they are not exclusive to individuals undergoing dialysis treatment.

No presence of HEV RNA was detected in any of the 225 patients included in the study, irrespective of their HEV serological profile. The lack of HEV RNA positivity among individuals receiving hemodialysis is a positive aspect due to the possible risks associated with the development of chronic hepatitis E, particularly in patients who have had organ transplantation or have pre-existing chronic liver disease. The prevalence studies conducted in the hemodialysis population have generally found that HEV RNA is not detectable [16,23,42,47]. This is likely because the generally asymptomatic clinical course of HEV infection does not typically prompt testing for hepatotropic viruses. Additionally, the short duration of viremia (with HEV RNA usually being detectable for about 4 weeks) [48] may also contribute to the absence of detection in these studies. 

The observed rise in the proportion of HEV-positive HD patients with increasing age, although not statistically significant (Figure 2), has been similarly documented in other research centers [6,41,42,47]. This phenomenon can be partially attributed to the tendency of dialysis research to primarily focus on the adult population (≥18 years old), rather than encompassing the entire age range typically observed in population studies. The data collected from the general population in the Plovdiv region revealed a clear association between the prevalence of HEV and age. The prevalence of HEV was found to be 3.53% in individuals aged 1–9 years, increasing to 19.23% in individuals aged over 60 years (*p* = 0.004) [31]. Publications on the subject provide heterogeneous information, from a pronounced age-dependence of HEV prevalence at the population level [49,50] and a corresponding lack thereof [47]. These disparities indicate that age alone is not a reliable indicator of positivity. A comprehensive study conducted in England examining stratification by cohorts based on birth period revealed that the likelihood of contracting HEV was not solely associated with age, but also with the exact time period in which individuals resided [50]. The aforementioned analysis demonstrated that the prevalence of HEV infection exhibited a notable peak during the mid-20th century, followed by a subsequent decline. The observation of a notable coincidence arises from the identification of a decrease in the proportion of positive cases exclusively within the 50–59 age group, accompanied by an overall increasing trend across different age groups (as depicted in Figure 2). Additionally, a decrease in the same age group has been observed among the general population residing in the Plovdiv region [32]. There is a larger likelihood that several factors not directly related to dialysis, such as occupational involvement, living environment, the availability of animal reservoirs carrying zoonotic HEV, and other factors, also contribute to the increased incidence of infection among the elderly. 

In both groups (HEV IgG seroprevalence and overall HEV seroprevalence), it was observed that the prevalence of positive outcomes was greater among males compared to females (*p* > 0.05) (Table 3). The observed disparity in our sample, with men comprising 56% and women comprising 44%, may potentially have implications; however, the statistical analysis did not yield a significant difference (*p* > 0.05). Several studies conducted in various hemodialysis centers have demonstrated an association between gender HEV prevalence [46], as well as its absence [22,42,43,51].

In a review article conducted by S. Hosseini-Moghaddam et al. from January 1980 to September 2009, it was observed that residing in rural areas as opposed to metropolitan areas appears to be a potential risk factor for HEV infection in HD patients [3]. In contrast to what was anticipated, the findings of our study revealed a divergent conclusion. Several writers have provided evidence of a relationship between the incidence of HEV and the individual’s place of residence, particularly in respect to geographical location [5,6]. The aforementioned association takes into account local customs such as dietary habits, outdoor activities, and involvement in agricultural practices.

No significant association was observed between the dietary patterns of individuals with HD, namely their consumption of pork, fish, and leafy greens, and the prevalence of HEV in both groups (Table 3), which is consistent with the similar findings of P. Jelicic et al. [16]. In a study conducted by Pisano et al., in Argentina [43], it was observed that there is an association between increased levels of HEV among patients undergoing hemodialysis and the consumption of fish, but not pork.

In the context of investigating the utilization of various water sources, a logistic regression analysis revealed a significant association between the consumption of bottled water and a reduced probability of HEV-positive cases (OR = 0.20, 95% CI: 0.04–0.99). This finding contrasts with the observations made by researchers in Croatia and Lebanon, who did not identify a similar relationship [15,16].

The characteristics that have been examined thus far were external to the hemodialysis facilities. The results of our univariate analysis indicate that there is no significant relationship between being HEV-positive or HEV-negative and characteristics such as age, sex, place of residence, and dietary habits (Table 3), as previously described.

Regarding predictors linked to underlying renal disease/dialysis treatment and HEV seropositivity, the univariate analysis demonstrated an association between the HEV IgG seroprevalence group and vascular access. The logistic regression analysis further substantiated the finding that patients with a permanent central venous catheter exhibited a higher likelihood of testing positive for HEV compared to patients with an AV fistula (OR = 12.26, 95% CI: 2.19–68.73). We did not find such an association in the available literature, but one has been reported for vascular access and HCV seroconversion in hemodialysis patients [52], indirectly pointing to a parenteral route of virus transmission. It is likely that vascular access in dialysis patients would be important for the nosocomial transmission of HEV, based also on the fact that low-molecular-weight heparins, used to maintain vascular patency and prevent thrombotic complications, are extracted from the porcine small intestine [53]. HEV RNA has been detected in the concentration of heparin derived from these animals, so the contaminated heparin with HEV infection may transmit HEV to HD patients [53].

The logistic regression analysis also confirmed the duration of hemodialysis treatment of more than 5 years as a predictor for HEV IgG positivity compared to individuals with a dialysis duration of less than a year. The findings of our study are supported by prior research undertaken by other authors [3,4,45], indicating the potential for transmission of HEV within hemodialysis units. However, the data are conflicting: in other studies, the duration of dialysis treatment was not associated with increased levels of HEV [22,23]. This discrepancy highlights the need for further focused investigation.

For the rest of the intra-dialysis factors, such as blood transfusions, operative interventions, HBsAg, and anti-HCV levels, their role in relation to HEV positivity has not been confirmed and is debatable in the scientific literature [3,4,7,15,23,45,54].

## 5. Conclusions

Our prospective seroepidemiological study among hemodialysis patients, using serological and molecular testing for HEV, is the first in Bulgaria and enables an accurate assessment of HEV prevalence. The binominal logistic regression analysis of available extra- and intra-dialysis risk factors confirmed the role of vascular access and a duration of dialysis treatment over 5 years as predictors significantly associated with increased risk for HEV IgG positivity, and the consumption of bottled water with lower levels of HEV IgG prevalence among hemodialysis patients. The inclusion of HEV markers in the screening of hemodialysis patients will enable early detection, follow-up, and, if necessary, treatment of cases of hepatitis E virus infection.

Limitations of the study: The main limitation of this study is the lack of a control group of age-matched healthy subjects to hemodialysis patients to assess whether the risk of HEV infection is higher in HD patients. Another significant disadvantage is that the serological analysis relied on a single commercial diagnostic test without verification from another or the conformation by the immunoblot test which may at least partly influence the serology results. This study would have a greater clinical value if, in parallel with the tests conducted for the presence of HEV markers, biochemical parameters, such as the level of liver enzymes, etc., were also examined. 

## Figures and Tables

**Table 1:** Summary of the demographic characteristics and evaluated risk factors, in relation to HEV positivity, among HD patients (*n* = 225).

Variable	Hemodialysis Patients (*n* = 225)
Age (year)	
Median	64
25th; 75th percentile	52.5; 71.5
Min–max	24–95
Age decades, *n* (%)	
18–49	44 (19.6)
50–59	43 (19.1)
60–69	61 (27.1)
≥70	77 (34.2)
Gender, *n* (%)	
Male	126 (56.0)
Female	99 (44.0)
M:F	1.3:1
Place of residence, *n* (%)	
Town	153 (68.0)
Village	72 (32.0)
Duration of hemodialysis (months)	
Median (25th percentile; 75th percentile)	48 (24; 72)
Duration of dialysis, *n* (%)	
≤1 year	60 (26.7)
2–5 years	91 (40.4)
>5 years	74 (32.9)
Vascular access, *n* (%)	
AV fistula	121 (53.8)
Permanent catheter	99 (44.0)
Vascular prosthesis	5 (2.2)
Previous kidney transplantation, *n* (%)	
Yes	9 (4.0)
No	216 (96.0)
Blood transfusion, *n* (%)	
Yes	132 (58.7)
No	93 (41.3)
History of surgical intervention, *n* (%)	
Yes	159 (70.7)
No	66 (29.3)
Diseases that led to hemodialysis, *n* (%)	
Autosomal dominant polycystic kidney disease	34 (15.2)
Chronic tubulointerstitial nephritis	34 (15.2)
Chronic glomerulonephritis	33 (14.7)
Chronic calculous pyelonephritis	25 (11.1)
Diabetic nephropathy	21 (9.3)
Hypertensive kidney disease	18 (8.0)
Bilateral hydronephrosis	10 (4.4)
Chronic nephritic syndrome	10 (4.4)
Other disease	40 (17.7)
Kidney disease in other family member, *n* (%)	
Yes	50 (22.2)
No	175 (77.8)
Hepatitis (jaundice) in the past *, *n* (%)	
Yes	22 (9.8)
No	203 (90.2)
Comorbidities **, *n* (%)	
Arterial hypertension	151 (67.1)
Secondary anemia	132 (58.7)
Ischemic heart disease	43 (19.1)
Heart failure	29 (12.9)
Diabetes	27 (12.0)
Nephrectomy	12 (5.3)
Oncological diseases	10 (4.4)
Liver cirrhosis	3 (1.3)
Traveled abroad in the last 3 months, *n* (%)	
Yes	1 (0.4)
No	224 (99.6)

* History of hepatitis (jaundice) in the past regardless of cause; ** the sum of the relative shares exceeds 100% (presence of patients with more than one concomitant disease).

**Table 2:** Distribution of patients according to the HEV, HBV, and HCV-positive serology in the three dialysis centers.

Variable	Center 1 (*n* = 80), %	Center 2 (*n* = 112), %	Center 3 (*n* = 33), %	*p*-Value
Overall HEV seroprevalence *, (*n* = 24)	7 (8.8%)	14 (12.5%)	3 (9.1%)	0.645
HEV IgG seroprevalence (*n* = 14)	4 (5%)	10 (8.9%)	0 (0%)	0.150
HBsAg (+), *n* (%)	5 (6.3%)	4 (3.6%)	4 (12.1%)	0.195
anti-HCV (+), *n* (%)	5 (6.3%)	8 (7.1%)	2 (6.1%)	0.937

* All specimens were negative for HEV RNA. The overall HEV seroprevalence group is based on all positive samples regardless of the type of HEV antibody marker (anti-HEV IgM and/or anti-HEV IgG).

**Table 3:** Variability of HEV seropositivity in relation to associated demographic characteristics and some of the risk factors among HD patients (univariate analysis).

Parameters	HEV IgG Seroprevalence (*n* = 14) *n* (%)	HEV Markers (−) (*n* = 201) *n* (%)	*p*-Value	Overall HEV Seroprevalence (*n* = 24) *n* (%)	HEV Markers (−) (*n* = 201) *n* (%)	*p*-Value
Age decades			0.473			0.462
18–49	2 (14.3)	41 (20.4)		3 (12.5)	41 (20.4)	
50–59	1 (7.1)	40 (19.9)		3 (12.5)	40 (19.9)	
60–69	4 (28.6)	54 (26.9)		7 (29.2)	54 (26.9)	
≥70	7 (50.0)	66 (32.8)			66 (32.8)	
Gender			0.918			0.808
Male	8 (57.1)	112 (55.7)		14 (58.3)	112 (55.7)	
Female	6 (42.9)	89 (44.3)		10 (41.7)	89 (44.3)	
Place of residence			0.770			0.753
Town	10 (71.4)	136 (67.7)		17 (70.8)	136 (67.7)	
Village	4 (28.6)	65 (32.3)		7 (29.2)	65 (32.3)	
Hemodialysis duration (yrs.)			0.522			0.306
≤1 year	2 (14.2)	56 (27.9)		4 (16.7)	56 (27.9)	
2–5 years	6 (42.9)	78 (38.8)		13 (54.2)	78 (38.8)	
>5 years	6 (42.9)	67 (33.3)		7 (29.2)	67 (33.3)	
Vascular access (Yes)			0.007			0.275
AV fistula	2 (14.3)	111 (55.2)		10 (41.7)	111 (55.2)	
Permanent catheter	12 (85.7)	85 (42.3)		14 (58.3)	85 (42.3)	
Vascular prosthesis	0 (0)	5 (2.5)		0 (0)	5 (2.5)	
Predictor variables						
Blood transfusion			0.938			0.687
Yes	8 (57.1)	117 (58.2)		15 (62.5)	117 (58.2)	
No	6 (42.9)	84 (41.8)		9 (37.5)	84 (41.8)	
Surgical intervention			0.458			0.149
Yes	11 (78.6)	139 (69.2)		20 (83.3)	139 (69.2)	
No	3 (21.4)	62 (30.8)		4 (16.7)	62 (30.8)	
Hepatitis infection in the past			0.203			0.327
Yes	0 (0)	21 (10.4)		1 (4.2)	21 (10.4)	
No	14 (100)	180 (89.6)		23 (95.8)	180 (89.6)	
Food consumption						
Pork			0.840			0.559
Yes	12(85.7)	176 (87.6)		22 (91.7)	176 (87.6)	
No	2 (14.3)	25 (12.4)		2 (8.3)	25 (12.4)	
Fish			0.226			0.054
Yes	13 (92.9)	160 (79.6)		23 (95.8)	160 (79.6)	
No	1 (7.1)	41 (20.4)		1 (4.2)	41 (20.4)	
Vegetable leafy greens			0.885			0.519
Yes	12 (85.7)	175 (87.1)		22 (91.7)	175 (87.1)	
No	2 (14.3)	26 (12.9)		2 (8.3)	26 (12.9)	
Water intake						
Tap water			0.548			0.687
Yes	7 (50.0)	117 (58.2)		15 (62.5)	117 (58.2)	
No	7 (50.0)	84 (41.8)		9 (37.5)	84 (41.8)	
Bottled water			0.268			0.975
Yes	8 (57.1)	143 (71.1)		17 (70.8)	143 (71.1)	
No	6 (42.9)	58 (28.9)		7 (29.2)	58 (28.9)	
Private water source			0.127			0.162
Yes	0 (0)	29 (14.4)		1 (4.2)	29 (14.4)	
No	14 (100)	172 (85.6)		23 (95.8)	172 (85.6)	
Contact with animals			0.714			1.000
Yes	4 (28.6)	67 (33.3)		8 (33.3)	67 (33.3)	
No	10 (71.4)	134 (66.7)		16 (66.7)	134 (66.7)	
HBsAg			0.792			0.570
Yes	1 (7.1)	11 (5.5)		2 (8.3)	11 (5.5)	
No	13 (92.9)	190 (94.5)		22 (91.7)	190 (94.5)	
anti-HCV			0.980			0.603
Yes	1 (7.1)	14 (7.0)		1 (4.2)	14 (7.0)	
No	13 (92.9)	187 (93)		23 (95.8)	187 (93.0)	

**Table 4:** Binominal logistic regression model for positive vs. negative result of HEV IgG seroprevalence.

Model	Unstandardized Coefficients		Wald	df	Sig.	Exp(B)	95% Confidence Interval for B	
	B	Std. Error					Lower Bound	Upper Bound
(Constant)	−4.16	1.97	4.42	1	0.036	0.02		
Age groups								
18–49 yrs. (baseline)								
50–59 yrs.	−1.92	1.36	1.98	1	0.159	0.15	0.01	2.12
60–69 yrs.	−0.28	1.01	0.08	1	0.785	0.76	0.11	5.48
≥70 yrs.	−0.18	0.95	0.04	1	0.852	0.84	0.13	5.41
Hemodialysis duration								
≤1 yr. (baseline)								
2–5 yrs.	0.92	0.94	0.97	1	0.325	2.52	0.40	15.91
>5 yrs.	1.90	0.97	3.84	1	0.049	6.67	1.01	44.37
Vascular access								
AV fistula (baseline)								
Vein prosthesis	−16.35	16 574.32	0.00	1	0.999	0.00	0.00	0.00
Permanent central vein catheter	2.54	0.88	8.38	1	0.004	12.72	2.27	71.20
Blood transfusion								
No (baseline)								
Yes	−0.69	0.68	1.02	1	0.31	0.50	0.13	1.92
Surgical intervention								
No (baseline)								
Yes	0.89	0.82	1.17	1	0.280	2.43	0.49	12.12
Food habits—consumption of pork								
No (baseline)								
Yes	−0.49	0.95	0.26	1	0.608	0.61	0.10	3.96
Food habits—consumption of fish								
No (baseline)								
Yes	1.06	1.17	0.82	1	0.366	2.88	0.29	28.54
Type of drinking water intake—tap water								
No (baseline)								
Yes	−1.25	0.85	2.16	1	0.142	0.29	.05	1.52
Type of drinking water intake—bottled water								
No (baseline)								
Yes	−1.69	0.85	3.91	1	0.048	0.19	0.04	0.99
Animal contact								
No (baseline)								
Yes	−0.37	0.78	0.23	1	0.635	0.69	0.15	3.17

## Data Availability

Data will be provided on request.
